# Plant-expressed cocaine hydrolase variants of butyrylcholinesterase exhibit altered allosteric effects of cholinesterase activity and increased inhibitor sensitivity

**DOI:** 10.1038/s41598-017-10571-z

**Published:** 2017-09-05

**Authors:** Katherine E. Larrimore, I. Can Kazan, Latha Kannan, R. Player Kendle, Tameem Jamal, Matthew Barcus, Ashini Bolia, Stephen Brimijoin, Chang-Guo Zhan, S. Banu Ozkan, Tsafrir S. Mor

**Affiliations:** 1 School of Life Sciences and Center for Immunotherapy, Vaccines, and Virotherapy, Biodesign Institute, Arizona State University, Tempe, AZ 85287-4501 USA; 2 Department of Physics and Center for Biological Physics, Arizona State University, Tempe, AZ 85287-1504 USA; 3 Department of Molecular Pharmacology and Experimental Therapeutics, Mayo Clinic, Rochester, MN 55905 USA; 4 Molecular Modeling and Biopharmaceutical Center and Department of Pharmaceutical Sciences, College of Pharmacy, University of Kentucky, Lexington, KY 40536 USA; 5 Present Address: Temasek Life Sciences Laboratory, National University of Singapore, Singapore, 117604 Singapore; 6 Present Address: Department of Botany, College of Letters and Sciences, University of Wisconsin Madison, Madison, WI 53706 USA; 7 Present Address: Department of Animal Science, College of Agriculture and Life Sciences, Cornell University, Ithaca, NY 14853 USA; 8 Present Address: ARUP Labs, Salt Lake City, UT 84108 USA

## Abstract

Butyrylcholinesterase (BChE) is an enzyme with broad substrate and ligand specificities and may function as a generalized bioscavenger by binding and/or hydrolyzing various xenobiotic agents and toxicants, many of which target the central and peripheral nervous systems. Variants of BChE were rationally designed to increase the enzyme’s ability to hydrolyze the psychoactive enantiomer of cocaine. These variants were cloned, and then expressed using the magnICON transient expression system in plants and their enzymatic properties were investigated. In particular, we explored the effects that these site-directed mutations have over the enzyme kinetics with various substrates of BChE. We further compared the affinity of various anticholinesterases including organophosphorous nerve agents and pesticides toward these BChE variants relative to the wild type enzyme. In addition to serving as a therapy for cocaine addiction-related diseases, enhanced bioscavenging against other harmful agents could add to the practicality and versatility of the plant-derived recombinant enzyme as a multivalent therapeutic.

## Introduction

The human serum enzyme butyrylcholinesterase (BChE) is a promiscuous enzyme capable of binding and/or hydrolyzing a diverse array of compounds including many natural and man-made toxicants of the central and peripheral nervous system, unlike the highly-selective, homologous enzyme, acetylcholinesterase (AChE)^1^. BChE is capable of counteracting the toxicity of various anticholinesterases by binding to them before they reach their targets in the nervous system. BChE is capable of detoxifying organophosphorous (OP) nerve agents like paraoxon, as well as acetylcholine receptor antagonists, and psychoactive plant alkaloids such as cocaine^2–5^. Exogenously-supplied BChE can augment the bioscavenging capacity of the endogenous enzyme and provide broad protection by sequestering the anticholinesterase agents^6–9^. Moreover, recombinantly-produced BChE variants with improved binding affinities and catalytic prowess can be created to improve on the parameters of the wild type (WT) enzyme.

In addition to improving BChE’s binding affinity toward anticholinesterase agents, the hydrolytic activity of human BChE (hBChE) against cocaine has also been a target for improvement. The catalytic activity of WT hBChE against cocaine is measurable, albeit slow, and provides one of the major detoxification pathways for the drug, generating non-psychoactive metabolites^10, 11^. Mutants of BChE have been rationally-designed, creating highly efficient recombinant cocaine hydrolases aimed toward an enzyme-based therapy to treat drug overdose and addiction^12–19^. When designing BChE-based cocaine hydrolase mutants, care was taken to ensure that their ability to hydrolyze the crucially important substrate, acetylcholine (ACh), was *not* significantly enhanced.

**Figure 1:**
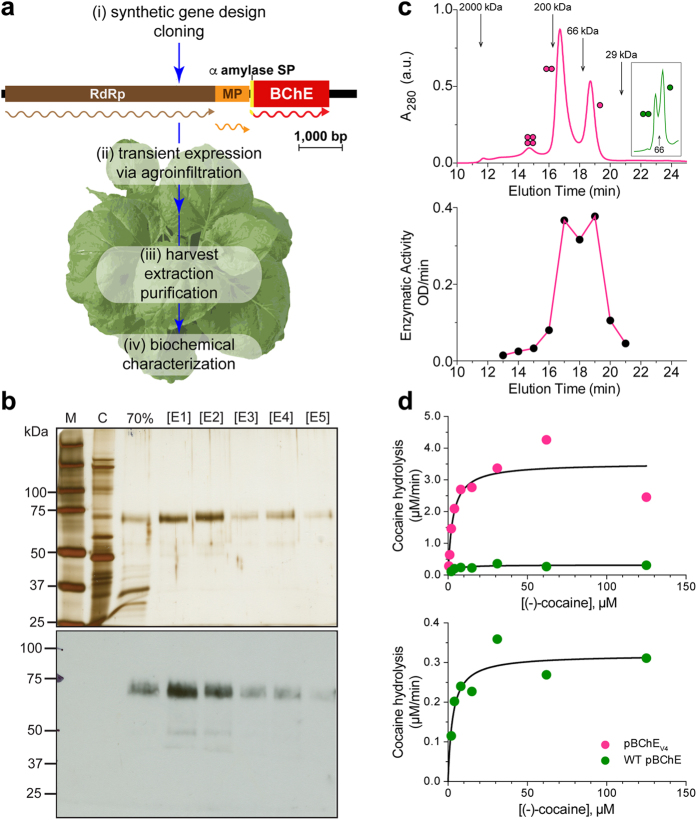
Plant production and biochemical characterization of a cocaine hydrolase variant of BChE. (**a**) Plant-based strategy for the production of BChE. (i) Plant-expression optimized synthetic genes encoding human BChE and variants thereof were cloned into the TMV-based MagnICON vector system, which recombines *in vivo* to yield a cell-to-cell-spreading replicon. (ii) WT *Nicotiana benthamiana* plants were infiltrated with agrobacteria harboring the MagnICON vectors (iii) and on peak accumulation day of the transiently-expressed recombinant enzymes, leaf material was harvested, homogenized and the enzymes were purified. Transient expression replicon: RpRd, RNA-dependent RNA polymerase; MP, movement protein gene; α, barley alpha-amylase signal peptide. Wavy lines represent the translation products of the replicon genes. (**b**) Purification of pBChE_V4_. Leaf extract from pBChE_V4_ –expressing plants was clarified by 70% (NH_4_)_2_SO_4_ precipitation then subject to ConA purification and eluted with stepwise increasing concentrations of methyl-α-D-mannopyranoside ([E1]-[E5]). Samples from these purification steps, protein size markers (M) and an un-infiltrated WT *N*. *benthamiana* extract control (C) were subject to SDS-PAGE followed by silver-staining (top) or BChE-specific immunoblotting (bottom). Lanes in respective gels were loaded based on equal enzymatic activity. (**c**) Oligomerization of pBChE_V4_. Purified preparation of pBChE_V4_ was analyzed by SEC-HPLC; fractions were monitored for total protein content (top) and pooled fractions (0.5 mL every 1 min) for enzymatic activity (bottom). Inset: fractionation pattern of WT pBChE. Molecular mass standards are indicated with arrows. (**d**) Enzymatic hydrolysis of (−)-cocaine by WT pBChE and pBChE_V4_. Purified samples of WT pBChE (green, 1.21 × 10^−1^ µM, upper and lower panel) and pBChE_V4_ (pink, 6.06 × 10^−4^ µM, upper panel). Curves represent nonlinear regression fitted to the Michaelis-Menten model (Equation 1). Fitting the data to the Radić model (substrate inhibition, Equation 2) does not result in a significantly better fit (based on the extra sum-of-squares F test; *p* > 0.12 and *p* > 0.78 for the mutant and WT enzymes, respectively).

A low-cost, sustainable, source of recombinant BChE must be readily available to produce clinically useful quantities of BChE mutants. Rapid and high level transient expression of foreign proteins in plants is needed to efficiently screen copious numbers of mutant variants, while maintaining the ability to ramp up production greatly when mutants of particular interest have been established. Mammalian expression systems have been used to produce cocaine hydrolase variants of BChE^20^, but such platforms can be difficult and expensive to scale up^21^. Plant-based recombinant protein production systems, in particular transient expression systems that make use of viral vectors (Fig. 1a), have advantages including reduced production costs, similar or cheaper downstream costs, as well as easy scalability^5, 22, 23^.

Our lab has previously shown that the tobacco relative *Nicotiana benthamiana* can serve as a source for clinically-relevant quantities of cocaine hydrolase variants of BChE^24, 25^. These highly efficient cocaine-metabolizing variants of BChE were designed with the goal of increasing catalytic efficiency of cocaine hydrolysis toward an anti-cocaine treatment. But how the newly introduced mutations affect the enzymes’ sensitivity to anticholinesterases and its kinetics with choline ester substrates remains unknown.

Here we report the complex kinetic behavior of the plant-derived cocaine hydrolase variants of BChE (pBChE) and their enhanced anticholinesterase scavenging ability. Using Dynamic Coupling Index (*DCI*) analysis we have evidence that the mutations allosterically affect the catalytic triad not only within a single subunit, but also propagate to neighboring subunits of the BChE oligomer.

## Results and Discussion

### Plant production of a recombinant cocaine-hydrolyzing human BChE variant

Several research groups have been working on rational re-design of BChE into a cocaine hydrolase^13, 15, 19, 26, 27^. The group led by Zhan used hybrid quantum mechanical/molecular mechanical (QM/MM) method-based predictions followed by validation through *in vitro* and *in vivo* experiments. This process provided evidence for a correlation between the measured catalytic efficiency of cocaine hydrolysis and the sum of the enzyme-substrate hydrogen-bonding distances within the first transition state. In successive papers Zhan *et al*. reported the further design of BChE variants with ever increasing catalytic efficiency^15–18, 28–32^.

We previously reported on several of these variants (see Methods for a list of variants and their specifically-modified residues as well as Supplementary Table S1) using the deconstructed tobacco mosaic virus (TMV)-based expression system in plants (Fig. 1a)^24^. This virus-assisted transient expression system exploits plant viral vectors deconstructed for the rapid, industrial-scale expression of foreign proteins^8, 9, 25^. In developing this technology, we focused on a variant, pBChE_V4_ (A199S/F227A/S287G/A328W/Y332G) reported to hydrolyze cocaine close to the upper limit set by substrate diffusion rates. Recently, another BChE variant with a 6^th^ mutation, P285A, was reported with further 2-fold better catalytic efficiency potentially bringing it to the diffusion-limited maximal theoretical ceiling^19^.

The pBChE_V4_ was purified as previously reported for its WT counterpart^8^. SDS-PAGE analysis of pBChE_V4_ revealed that it resolved with an apparent molecular mass of ~65–70 kDa. This is similar to previously described plant-derived BChE variants and slightly smaller than the ~85 kDa human BChE monomer, likely due to differences in glycosylation (Fig. 1b)^8, 33^. When highly-purified pBChE_V4_ was subjected to SEC-HPLC, about two thirds was dimeric (Fig. 1c). Most of the remainder were monomers, but low amounts of tetramers were also detected (Fig. 1c). Similar preparations of the WT enzyme, obtained through transient expression using the MagnICON system, showed inverse proportions of monomers and dimers (Fig. 1c inset). Interestingly, stable expression of WT enzyme in transgenic plants results in a substantial tetramer fraction^8, 9^.

The plant-derived pBChE_V4_ was examined closely for its ability to hydrolyze (−)-cocaine (Fig. 1d) and was found to have >2000-fold improved catalytic efficiency against that substrate (*k*_cat_/K_M_ = 1.9 × 10^9^ M^−1^ min^−1^) compared with the WT plant-derived enzyme (*k*_cat_/K_M_ = 9.0 × 10^5^ M^−1^ min^−1^). The higher efficiency is mostly due to a large increase in *k*_cat_ of pBChE_V4_ as compared to WT pBChE (5805 min^−1^ *vs* 2.6 min^−1^, respectively) with nearly identical affinity to the substrate (K_M_ = 3.0 µM *vs* K_M_ = 2.9 µM, respectively). The catalytic efficiency of the plant-derived variant and its improvement over WT BChE are in agreement with reports of this same variant derived from other sources such as human embryonic kidney-293F cells^34^.

### Cocaine hydrolase variants of BChE exhibit altered allosteric effects

The specific residues changed on the road to BChE-based cocaine hydrolases included those at the bottom of the catalytic gorge near the π-cation binding site (A328) and in the peripheral anionic site (Y332)^13, 14^. Catalytic activity against (−)-cocaine was further improved through additional mutations to the oxyanion hole (A199)^32^, entrance to the gorge (S287)^15^ and non-active site residues participating in H-bonding (F227)^29^. Together, these changes result in increased catalytic efficiency against (−)-cocaine and potentially affect the enzyme’s interactions with other substrates and ligands. Indeed, preliminary results with crude preparations revealed such effects (presented in The XI^th^ International Meeting on Cholinesterases, Kazan, Russia, June 4–9, 2012 “Plant-produced butyrylcholinesterase variants as versatile bioscavengers”). It was, therefore, of interest to determine if there were other such allosteric effects on the function of several plant-derived BChE variants.

**Figure 2:**
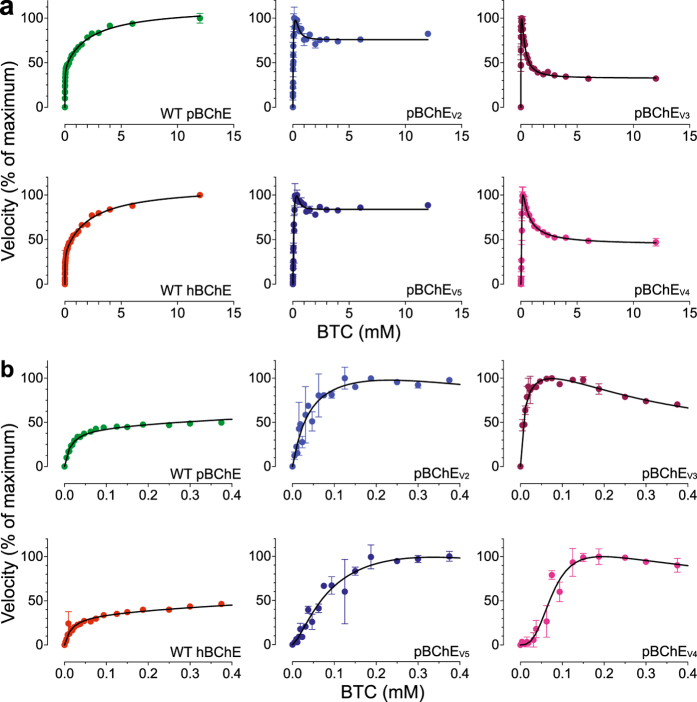
BTC hydrolysis by WT hBChE, WT pBChE, and pBChE_V2-5_. (**a**) Reaction rates are plotted against substrate concentration (mean ± SEM). Plots in (**b**) zoom in on the low range of substrate concentrations. The 100% values and the goodness of fit values are as follows: WT hBChE, 100% = 1.57 ± 0.04 nmol/min, Equation (2), R^2^ = 0.98; WT pBChE, 100% = 1.21 ± 0.07 nmol/min, Equation (2), R^2^ = 0.99; pBChE_V2_, 100% = 0.79 ± 0.10 nmol/min, Equation (3), R^2^ = 0.83; pBChE_V3_, 100% = 0.96 ± 0.01 nmol/min, Equation (3), R^2^ = 0.95; pBChE_V4_, 100% = 8.9 ± 0.6 nmol/min, Equation (3), R^2^ = 0.95; pBChE_V5_, 100% = 8.1 ± 0.3 nmol/min, Equation (3), R^2^ = 0.95.

**Figure 3:**
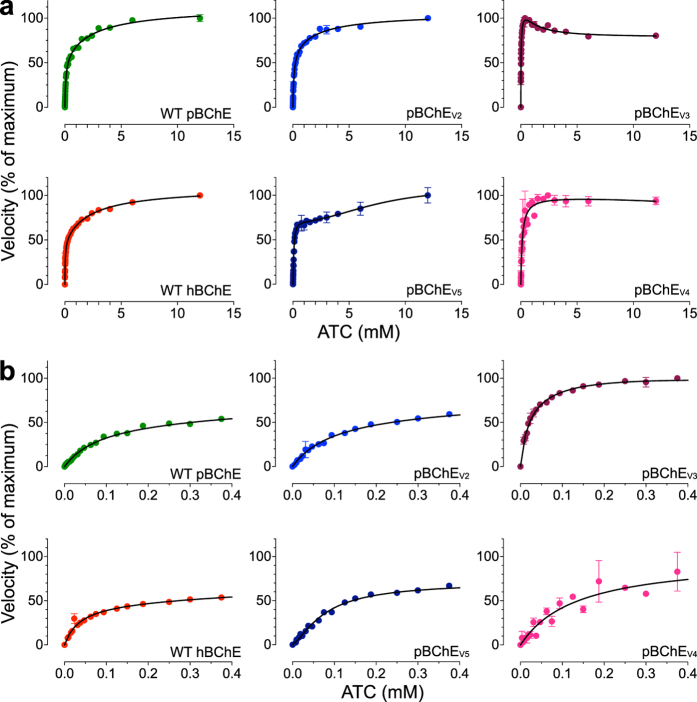
ATC hydrolysis by WT hBChE, WT pBChE, and pBChE_V2-5_. (**a**) Reaction rates are plotted against substrate concentration (mean ± SEM). Plots in (**b**) zoom in on the low range of substrate concentrations. The 100% values and the goodness of fit values are as follows: WT hBChE, 100% = 0.97 ± 0.04 nmol/min, Equation (2), R^2^ = 0.99; WT pBChE, 100% = 3.0 ± 0.1 nmol/min, Equation (2), R^2^ = 1.00; pBChE_V2_, 100% = 4.7 ± 0.1 nmol/min, Equation (2), R^2^ = 0.99; pBChE_V3_, 100% = 1.39 ± 0.00 nmol/min, Equation (3), R^2^ = 0.98; pBChE_V4_, 100% = 2.6 ± 0.1 nmol/min, Equation (1), R^2^ = 0.94; pBChE_V5_, 100% = 12.0 ± 0.7 nmol/min, Equation (3), R^2^ = 0.99.

To rule out artifacts from our novel expression system, we first compared WT human plasma-derived (hBChE) to pBChE. The Michaelis-Menten constant (K_M_) of WT hBChE and WT pBChE was determined with the substrate, butyrylthiocholine. Nonlinear regression analysis showed values of 16.8 ± 2.9 μM and 14.6 ± 1.4 μM respectively, similar to previous reports^30^ and essentially identical to each other (Figs 2 and 3, Table 1). WT hBChE and WT pBChE also exhibited similar turnover numbers (*k*_*cat*_ *= *2.6 × 10^4^ min^−1^ and *k*_cat_ = 2.6 × 10^4^ min^−1^, respectively) and catalytic efficiencies with the substrate analog butyrylthiocholine (BTC, *k*_*cat*_/K_M_ = 1.6 × 10^9^ M^−1^min^−1^ and *k*_*ca*t_/K_M_ = 1.8 × 10^9^ M^−1^ min^−1^, respectively; Fig. 2, Table 1).

**Table 1:** Catalytic activity of WT BChE and cocaine hydrolase variants against butyrylthiocholine and acetylthiocholine.

Substrate		WT hBChE	WT pBChE	pBChE_V2_	pBChE_V3_	pBChE_V4_	pBChE_V5_
**BTC**	**Kinetic behavior**	Substrate activation	Substrate activation	Modified Hill	Modified Hill	Modified Hill	Modified Hill
	***k***_**cat**_/**K**_**M**_ **(M** ^**−1**^ **min** ^**−1**^ **)**	1.6 × 10^9^	1.8 × 10^9^	1.8 × 10^7^	4.9 × 10^7^	3.4 × 10^7^	3.2 × 10^8^
	***k***_**cat**_ **(min)**	26241.2	25992.7	664.6	463.0	2806.0	27440.1
	**K**_**M**_ **(µM)**	16.8 ± 2.9	14.6 ± 1.4	37.1 ± 21.8	9.5 ± 3.2	83.0 ± 21.5	86.2 ± 27.7
	**K**_**ss**_ **(mM)**	2.2 ± 0.4	2.3 ± 0.3	0.4 ± 0.3	0.3 ± 0.1	0.4 ± 0.4	0.4 ± 0.2
	**b** ^**a**^	3.1 ± 0.2	2.5 ± 0.1	0.6 ± 0.2	0.3 ± 0.1	0.3 ± 0.2	0.7 ± 0.1
	**n** ^**b**^	n.a.	n.a.	1.1 ± 0.3	1.1 ± 0.2	2.8 ± 0.6	1.0 ± 0.2
	**x** ^**b**^	n.a.	n.a.	2.3 ± 2.0	2.3 ± 0.3	1.1 ± 0.6	1.5 ± 0.3
	***R*** ^***2***^	0.98	0.99	0.83	0.95	0.95	0.95
**ATC**	**Kinetic behavior**	Substrate activation	Substrate activation	Substrate activation	Modified Hill	Michaelis-Menten	Modified Hill
	***k***_**cat**_/**K**_**M**_ **(M** ^**−1**^ **min** ^**−1**^ **)**	2.5 × 10^8^	9.6 × 10^7^	3.7 × 10^7^	9.1 × 10^6^	8.3 × 10^6^	2.1 × 10^8^
	***k***_**cat**_ **(min)**	9185.0	8490.1	3756.3	243.2	1093.3	16154.1
	**K**_**M**_ **(µM)**	36.7 ± 3.8	89.2 ± 7.8	101 ± 13	26.8 ± 4.8	132 ± 13	77.0 ± 4.5
	**K**_**ss**_ **(mM)**	2.3 ± 0.4	2.7 ± 0.5	2.4 ± 1.0	1.2 ± 0.4	n.a.	7.6 ± 3.3
	**b**	2.2 ± 0.1	1.9 ± 0.1	1.6 ± 0.1	0.7 ± 0.1	n.a.	1.6 ± 0.3
	**n**	n.a.	n.a.	n.a.	0.9 ± 0.1	n.a.	1.4 ± 0.1
	**x**	n.a.	n.a.	n.a.	1.4 ± 0.5	n.a.	2.3 ± 1.1
	***R*** ^***2***^	0.99	1.0	0.99	0.98	0.94	0.99

^a^When b > 1, enzyme is exhibiting substrate activation; when b < 1, enzyme is exhibiting substrate inhibition; if b = 1, the enzyme is following Michaelis-Menten kinetics.

^b^n and x represent the Hill coefficients. See Scheme 2 of Supplementary Fig S1 online and Equation 3. Positive cooperativity is observed when either n > 1 or x > 1. Negative cooperativity is observed when n < 1 or x < 1.

Catalytic efficiency of pBChE_V2_ (F227A/S287G/A328W/Y332A), pBChE_V3_ (A199S/S287G/A328W/Y332G), and pBChE_V4_ (A199S/F227A/S287G/A328W/Y332G) toward BTC was reduced 100-, 37- and 5-fold, respectively, mostly due to a large reduction in the turnover number but also to small changes in the K_M_. An even larger drop (~1000-fold) was observed in the catalytic efficiencies of pBChE_V3_ and pBChE_V4_ toward the substrate analog acetylthiocholine (ATC), but not in the case of pBChE_V2_, which dropped only 3-fold. Of note, the catalytic efficiency of pBChE_V5_ (F227A/S287G/A328W/Y332G) toward both substrates remained equal to the WT enzyme (Table 1).

Human AChE and BChE exhibit characteristic allosteric effects due to low-affinity substrate binding at the “peripheral site” (P-site) positioned near the entrance to the catalytic gorge^35, 36^. Despite the close homology between the two cholinesterases, their substrates exert opposite allosteric effects. AChE is inhibited by ACh concentrations above 5 mM, but BChE is *stimulated* by similar concentrations of ACh and BCh and their thioester analogues (ATC and BTC, Figs 2 and 3)^30, 35, 37–41^. This remains true regardless of the source of the enzyme, as both WT hBChE and WT pBChE exhibited typical substrate activation against BTC and ATC (Figs 2 and 3, Table 1)^8^. A simple modification of the Michaelis-Menten model (Equation 1) results in an adequate steady-state description of the phenomena of substrate activation and inhibition in WT cholinesterases (Equation 2, Scheme 1 in Supplementary Fig. S1)^39^.

In striking contrast, hydrolysis of BTC by pBChE_V3_ and pBChE_V4_ revealed partial substrate inhibition (Fig. 2) reminiscent of the kinetics of human AChE with ACh as was previously reported for native and plant-derived human enzyme^40, 41^. But this inhibition (~40%) was much weaker than that exhibited by AChE (>90%), and their respective peak activities were reached at BTC concentrations of approximately 70 µM and 230 µM respectively (Fig. 2).

Even more complex enzymatic behavior was exhibited by pBChE_V2_ and pBChE_V5_, which differ from each other only by, respectively, an alanine or a glycine residue at position 332 (Fig. 2). BTC at concentrations higher than approximately 125 to 375 µM had more limited inhibitory effect on these variants (about 20%, Fig. 2) as compared to pBChE_V3_ and pBChE_V4_. Interestingly, at still higher substrate concentrations (>2 mM) very slight but highly reproducible substrate activation re-appeared (Fig. 2).

Hydrolysis of the smaller substrate ATC also revealed differences between the four variants. In all tested variants, ATC has much weaker inhibitory effect on its hydrolysis. In fact, pBChE_V2_ and pBChE_V5_, which were somewhat inhibited by high concentrations of BTC, were clearly activated by high ATC concentrations, as was the case of WT hBChE or WT pBChE (Fig. 3, Table 1). Still, pBChE_V3_ was inhibited at high concentrations of ATC (but not to the extent that BTC provoked), while pBChE_V4_ was not allosterically affected by the smaller substrate, exhibiting a hyperbolic Michaelis-Menten kinetic profile (Fig. 3).

**Table 2:** Inhibition of BTC hydrolysis activity.

	Paraoxon		Iso-OMPA		Neostigmine		BW284c51	
WT hBChE	−8.04 ± 0.04	(0.9)	−4.65 ± 0.03	(0.8)^**^	−6.79 ± 0.02	(1)^****^	−3.86 ± 0.03	(2)^****^
WT pBChE	−8.11 ± 0.03	(1)	−4.77 ± 0.03	(1)	−6.64 ± 0.01	(1)	−3.55 ± 0.05	(1)
pBChE_V2_	−9.69 ± 0.06	(38)^****^	−6.45 ± 0.05	(48)^****^	−7.75 ± 0.04	(13)^****^	−3.82 ± 0.11	(2)^*^
pBChE_V3_	−8.65 ± 0.07	(3)^****^	−4.45 ± 0.10	(0.5)^***^	−6.69 ± 0.05	(1)	−4.16 ± 0.10	(4)^****^
pBChE_V4_	−8.89 ± 0.05	(6)^****^	−4.87 ± 0.05	(1)	−6.81 ± 0.03	(1)^****^	−4.62 ± 0.09	(12)^****^
pBChE_V5_	−9.77 ± 0.05	(46)^****^	−6.39 ± 0.06	(42)^****^	−7.88 ± 0.03	(17)^****^	−3.31 ± 0.11	(1)^*^

Log IC_50_ values ± SEM of various anticholinesterase inhibitors versus WT hBChE, WT pBChE, and pBChE_V2-5_. Fold increase in sensitivity relative to WT pBChE is shown in parentheses. Concentration of BChE variants was ~5 mU. *p < 0.05. **p < 0.01. ***p < 0.001. ****p < 0.0001.

Chen *et al*. found similar results regarding BTC for one of their cocaine hydrolyzing variants^42^. Their mutant, hCocH, is a mammalian cell-derived equivalent of pBChE_V4_. They suggested that the change from substrate activation to substrate inhibition was due to destabilization of the rate-limiting step’s transition state when a second substrate molecule binds in the peripheral site. This is plausible but remains speculative at this point.

The complex kinetic behavior of certain variants is reflected by the relatively poor fit between experimental data and the standard model for BChE and AChE’s allosteric effects (Equation 2, Scheme 1 in Supplementary Fig. S1). A closer look at hydrolysis rates at low BTC concentrations (Figs 2b and 3b) showed a sigmoidal pattern as BTC concentrations rise. Sigmoidal behavior is characteristic for homo-oligomeric enzymes that exhibit cooperative binding of substrate molecules. Both BChE and AChE are oligomeric, tetramers and dimers being most common *in vivo*. But the common view is that oligomerization status does not affect the enzymatic properties of either enzyme^43–45^. BChE purified from transgenic plants is about 50% tetrameric^8, 9^, while TMV-assisted transient-expression in plants yields a mixture of monomers and dimers with few tetramers (Fig. 1c)^46^. As will be explored further, it is possible that mutations introduced into BChE to improve its activity toward cocaine also affected subunit interactions, which in turn made the enzyme behave cooperatively. Nonetheless, even monomeric enzymes with multiple substrate binding-sites, like all cholinesterases, can also exhibit cooperative (or anticooperative) binding. We found that including Hill coefficients describing cooperativity (or anticooperativity) into the standard analysis of uncompetitive inhibition, provides an adequate model to describe the behavior of the BChE variants against BTC and ATC (Scheme 2 in Supplementary Fig. S1). While the molecular mechanism is not yet established, the suggested model yields an estimate for factors that are assumed to be negligible for the WT enzymes (Scheme 2 in Supplementary Fig. S1). Specifically, the model anticipates the possibility that binding of one substrate molecule at either the peripheral or the active site may alter the binding of a second molecule in the other site. The Hill coefficients (Table 1) demonstrate weak positive cooperativity, which would be particularly important at low substrate concentrations. At higher substrate concentrations, effects on *k*_cat_ are more prominent and result in the observed substrate inhibition (against BTC) and activation (against ATC). A non-equilibrium analysis of the interactions between the peripheral site and the active site, similar to the one offered by Rosenberry^47^, should provide further insight into the mechanism involved here.

### Inhibition analysis

The mutations rendering enzyme variants with the ability to efficiently hydrolyze (−)-cocaine had profound allosteric effects on cholinesterase activity and our data suggest similar effects of those mutations on sensitivities to various anticholinesterases. To test this possibility we studied representatives of several important cholinesterase inhibitor classes including two OPs (paraoxon and Iso-OMPA), a carbamate (neostigmine) and an AChE-specific bisquaternary inhibitor (BW284c51). To this end, BTC hydrolysis was analyzed following a 30-minute incubation with the inhibitors.

**Figure 4:**
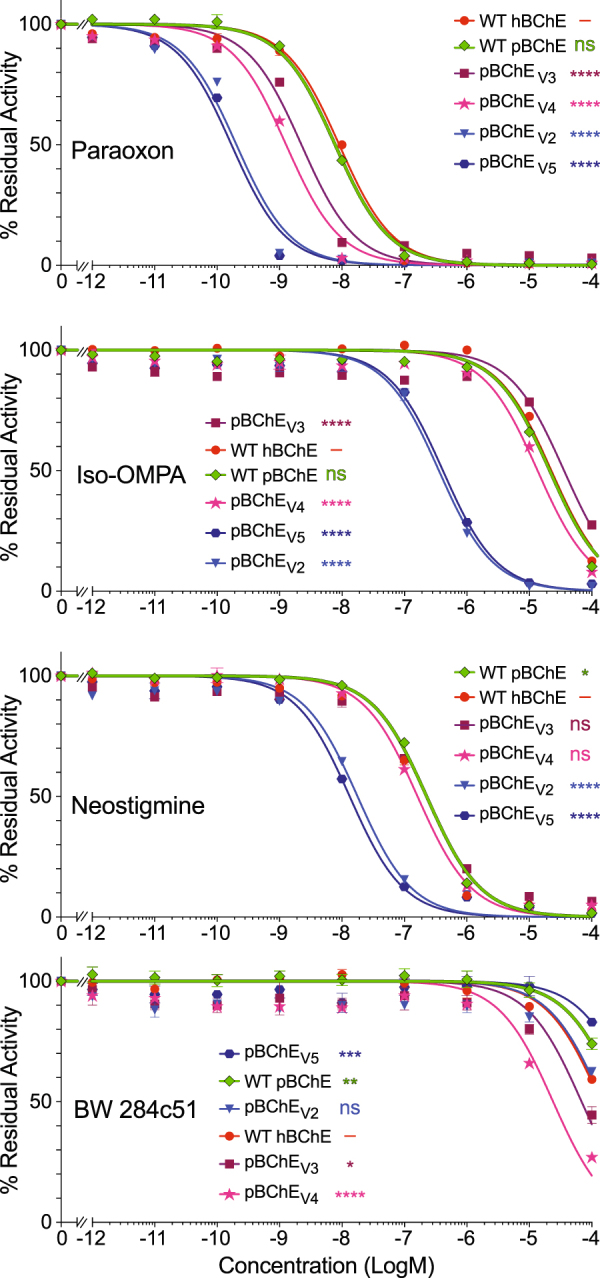
Inhibition profiles of WT hBChE, WT pBChE and pBChE_V2-5_. Residual BTC hydrolytic activity (mean ± SEM) with the indicated concentrations of paraoxon and Iso-OMPA (OP inhibitors), neostigmine bromide (a carbamate inhibitor) and BW (an AChE-specific bis-quaternary inhibitor). The legends in each panel list the traces in order of decreasing IC50. Plots of variants are compared to the human plasma-derived enzyme. ns, no statistical difference; *p < 0.05; **p < 0.01; ***p < 0.001; ****p < 0.0001.

Compared to the WT enzyme (either plasma- or plant-derived), pBChE_V2_ and pBChE_V5_ had dramatically increased sensitivity, reflected in decreased IC_50_ values. This was true for all tested anticholinesterase agents except for the AChE-specific inhibitor BW284c51 (Fig. 4, Table 2). In fact, each of the variants were 40–50 fold more sensitive to both OPs paraoxon and Iso-OMPA than the WT enzyme (p < 0.0001). In respect to neostigmine, the variants were also more sensitive than WT BChE but with smaller differences (10–20 fold). Similarly, increased sensitivities were observed in an earlier plant-derived cocaine-hydrolyzing variant pBChE_V1_ (A328W/Y332A) previously described by Geyer *et al*.^25^.

Higher-than WT sensitivities to paraoxon were also seen with pBChE_V3_ and pBChE_V4_ but the increase was not as dramatic as in the other two variants (3–7 fold, Fig. 4, Table 2). The inhibition rate constants (k_i_) for inhibition by paraoxon of WT pBChE and pBChE_V4_ were 3.14 × 10^6^ and 1.7 × 10^7^ M^−1^ min^-1^ respectively. On the other hand, sensitivities to the other OP, iso-OMPA, and to neostigmine were near WT levels.

Particularly interesting was the unexpected and small but statistically significant increase in sensitivity of pBChE_V3_ and pBCHE_V4_ toward the AChE-specific bisquaternary inhibitor BW284c51 as compared to pBChE (4- and 12-fold greater than WT, respectively). Thus, these variants have another AChE-like attribute besides substrate inhibition. The inhibitor spans the length of the catalytic gorge, from the P-site at the surface of the protein to the active site at the bottom of the gorge^48^. The only mutation common to both pBChE_V3_ and pBCHE_V4_ and absent from the other two variants is A199S. This alanine residue is conserved in both AChE and BChE and is adjacent to the catalytic triad’s serine residue (S198). The equivalent position in AChE was not identified in previously published research as relevant for the interaction with BW284C51. The hydroxyl of the serine residue may contribute a new H-bond to interact with the central carbonyl of BW284c51. However, without further data we cannot rule out contributions to the increased sensitivity through direct and/or allosteric effects of other residue changes in addition to the A199S mutation.

The substantially enhanced sensitivity of the cocaine hydrolases, especially pBChE_V2_ and pBChE_V5_ to the OP poisons, has important potential implications for detoxifying these harmful substances. One approach to detoxification is to supply WT BChE to scavenge nerve agents. Another approach is to enhance BChE’s binding affinity to anticholinesterase agents and create a more effective bioscavenger. Excitingly, though perhaps not surprising, mutations to BChE intended to enhance cocaine hydrolysis have altered the binding affinity of the cocaine hydrolases toward anti-cholinesterase inhibitors. The enhanced scavenging for OP nerve agents by the cocaine hydrolyzing variants suggests further development for dual use of the biologics.

### Dynamic coupling index (*DCI*) analysis predicts allosteric coupling between the pentavalent mutations of pBChE_V4_ and its active site

While all four variants exhibit novel enzymatic properties, the superior efficiency of cocaine hydrolysis of pBChE_V4_ compared to other variants (Fig. 1d, unpublished data and Zheng *et al*.^16, 19^), prompted further investigation of this variant. Specifically, we reasoned that the altered substrate preference, hydrolysis kinetics, and inhibitor sensitivity suggest that the mutated positions in pBChE_V4_ (A199S/F227A/S287G/A328W/Y332G), all but one being quite distal to the active site, may be allosterically linked to the catalytic locus. To test this hypothesis we used a recently developed metric, the “Dynamic Coupling Index” (*DCI*)^49^ that identifies residues exhibiting significant fluctuation upon perturbation of functionally important loci including the active catalytic site and other substrate binding sites in the protein^50^.

Using *DCI* analysis, we identified positions that dynamically couple to residues of the catalytic triad, i.e. S198, E325 and H438. According to this analysis, positions exhibiting high *DCI* values present residues that are dynamically linked to the active site despite being far away from the catalytic residues.

**Figure 5:**
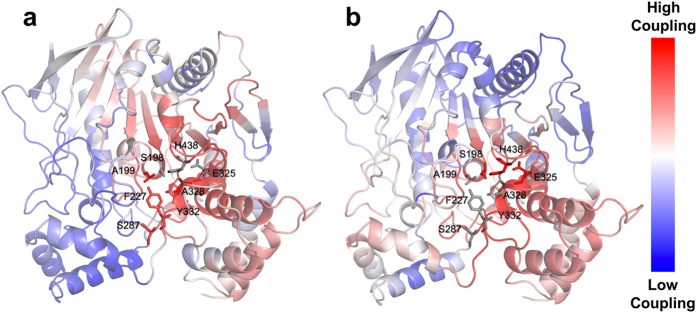
% *DCI* profile of WT hBChE. The % *DCI* profiles for hBChE are color-coded in a cartoon diagram from a spectrum of red-white-blue (red -highest, blue -lowest coupling to perturbation locations). (**a**) Upon perturbation of catalytic residues (S198, E325, and H438 shown as grey sticks) the five mutation positions (A199, F227, S287, A328, and Y332 shown as red sticks) shows high coupling (high % *DCI* values). (**b**) Upon perturbation of five mutation positions (A199, F227, S287, A328, and Y332 shown as grey sticks) the catalytic residues (S198, G325, and H438 shown as red sticks) shows high coupling (high % *DCI* values).

In Fig. 5a, the % *DCI* values for human BChE upon perturbation of the three catalytic residues are color-coded within a spectrum of red-white-blue (from highest to lowest respectively). It appears that the five mutated positions of pBChE_V4_ variant (A199S, F227A, S287G, A328W and Y332G) are highly coupled to the catalytic triad. Conversely, a reciprocal analysis of perturbing the five mutated positions and measuring % *DCI* values for other residues show that the catalytic triad’s residues are highly coupled to these five mutated positions (Fig. 5b). This reaffirms our hypothesis concerning dynamic interplay between these mutated positions and catalytic residues. Moreover, the strong dynamic coupling between mutational sites and the catalytic site suggests that mutations alter the conformational dynamics of the enzyme, leading to changes in enzymatic function.

The changes in catalytic properties of BChE variants can be partially attributed to the direct allosteric effect of peripheral amino-acid substitution on the catalytic triad suggested by *DCI* analysis (Fig. 5). Our results also raise the possibility that such mutations affect the interactions between enzyme subunits – specifically they may lead to increased enzymatic cooperativity (Figs 2 and 3).

### Dynamic flexibility index (*DFI*) analysis predicts global flexibility changes upon introduction of mutations

To further substantiate our hypothesis and provide mechanistic insights on how these five mutations lead to changes in enzymatic behavior, we explored the conformational dynamics of the WT and the pBChE_V4_ variant using a dynamic flexibility index (*DFI)*. *DFI* computes the fluctuation response of a given position to the perturbations that occur at different parts of the protein using linear response theory, capturing the multi-dimensional effects when the protein structure is displaced out of equilibrium for example when interacting with small molecules or other cellular constituents. *DFI* allows us to identify and map flexible and rigid positions in the structure^51, 52^. *DFI* can be considered a measure of the local conformational entropy of a given position within the set of interactions governed by the 3D fold of the protein due to its ability to probe the conformational space of a protein at the residue level. For example, we recently used *DFI* to provide mechanistic insights about emergence of new functions during the evolution of several protein families^53, 54^ and to explain the molecular basis of single-nucleotide polymorphisms associated with genetic-diseases^49, 52, 55^.

**Figure 6:**
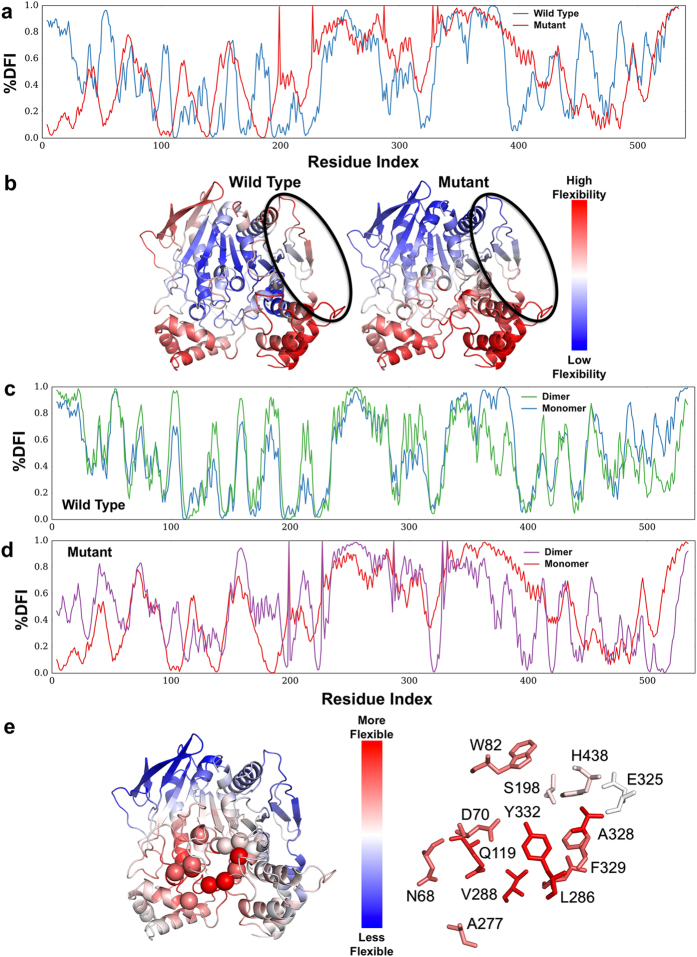
% *DFI* profile of WT hBChE and pentavalent mutant. (**a**) The % *DFI* profiles of WT BChE (blue) and BChE_V4_ (x-axis – residue numbers, y-axis – % *DFI* values at each position). (**b**) Color-coded structure diagrams depicting the % *DFI* values at each position. The circled regions are part of the monomer-monomer contact region (V377, D378, T457, K458, A459, I462, Y500, R509, M511, T512, K513, R514, L515). (**c**) The % *DFI* profiles of monomeric (blue) and dimeric (green) WT BChE. (**d**) The % *DFI* profiles of monomeric (red) and dimeric (purple) BChE_V4_. (**e**) Color-coded structure diagrams depicting the values of % *DFI* differences between the dimeric forms of WT BChE and BChE_V4_ at each position. The red-white-blue code reveals loci with increased flexibility (shades of red), decreased flexibility (shades of blue) or no change (white) in the mutant dimer vs. the WT dimer.

We measured the *DFI* values of residues for WT hBChE and pBChE_V4_, and the % *DFI* profiles shows us the flexibility of the proteins in ranking order (Fig. 6a). Examining the flexibility of the monomer-monomer contact (binding) interfaces (Fig. 6b), it appears that the dimerization surface of pBChE_V4_ is less flexible in comparison with the WT counterpart. Rigidified monomer-monomer interface is often associated with increased affinity^56, 57^. The association constant for dimerization depends on the entropic cost at the binding interface: dimerization causes the binding interface to be more rigid and is therefore causing a decrease in entropy (negative entropy change associated with dimerization i.e. ΔS_dimerization_ < 0). Because the entropy level associated with the WT contact surface is higher than in the mutant (i.e., the former is more flexible than the latter). Hence the entropic cost of dimerization is higher in WT than in the mutant (i.e. ΔS_dimerization_ of WT is more negative than that of the mutant). These results support our observation that preparations of pBChE_V4_ have higher proportion of dimers as compared to pBChE, which is mostly monomeric.

The oligomerization of WT BChE is usually not regarded as affecting the enzymatic properties of the enzyme. However, the sigmoidal nature of the enzyme kinetics observed here in the mutant variants (Figs 2 and 3) suggests a degree of cooperativity. If this is the case we should see that dimerization induces new conformational dynamics in the mutant but less so in the WT. To test this possibility, we explored how dimerization may affect the dynamics of each monomeric subunit in WT and the mutant using *DFI* analysis. The five residue substitutions are introduced into the Elastic Network Model (ENM) model (see Methods) at the core of the *DFI* analysis as changes in the spring constants of the harmonic oscillators interconnecting the alpha-carbons of the adjoining amino-acids (Supplementary Fig. S2). In other words, since the mutations introduced local destabilization around the mutational sites, we modeled this effect as decreased spring constants for the interactions of the mutational positions (i.e. weakened harmonic interactions of the mutational sites).

With this approach, we can predict global changes in flexibility upon introduction of mutations. The local disruption due to the mutations not only introduce enhanced flexibilities at the mutational sites, but can create a global flexibility change in all positions (i.e. change in *DFI* profile) due to network of interactions. In fact, it appears that the change in the flexibility of one region is compensated by the changes in flexibility of other regions. As could be expected based on the well-documented lack of cooperativity in BChE upon its oligomerization, the *DFI* profile of the WT hBCHE subunit in monomeric and dimeric form are quite similar (Fig. 6c). On the other hand, in the case of the BChE_V4_ mutant, the *DFI* profile of subunit in the dimer form differs notably from that in monomeric form (Fig. 6d). This change suggests that dimerization induces new conformational dynamics. Interestingly, when we map the localized differences in the % *DFI* values between mutant and WT, we observed that the mutations lead to enhanced flexibility near the gorge site in the dimer, but less so in the monomer (Fig. 6e). The peripheral anionic site (D70, N68, Q119, A227), cation- π domain (W82, A328), acyl pocket (L289 V288), and phenothiazine ring site (Y332, F329) exhibited increased flexibility upon mutations, while the rigid profile of the catalytic triad (S198, E235, H438) did not change.

The *DFI* analysis suggests that compared to the WT, BChE_V4_ should have a better propensity to dimerize and that within the mutant dimer there is an increase in flexibility near the gorge (Fig. 6e). We propose that changes in flexibility might facilitate propagation of conformational changes from one subunit to the other. Thus, at low substrate concentrations, binding of a substrate molecule on one of the subunits might positively affect substrate binding and/or turnover at the catalytic gorge of the other subunit, explaining the sigmoidal kinetic observed at low substrate concentrations (Figs 2b and 3b). At higher substrate concentrations, allosteric effects within each subunit may lead to inhibition countering the cooperative enhancement and explaining the observed partial substrate inhibition (Figs 2a and 3a). While speculative, this suggested mechanistic explanation raises several predictions that will be tested by further experimentation and simulation including substrate docking.

## Conclusions

The biochemical characterization of the plant-derived cocaine hydrolases reported here offers not only a better understanding of a novel anti-cocaine treatment, but also possible protection from potent pesticides and other anticholinesterase agents. The outcomes demonstrate the practicality and versatility of plant-derived recombinant enzymes as potential multivalent biologics. As new mutations are being found to establish even more efficient cocaine hydrolases, the results reported here point toward the importance of testing these enzymes for their altered kinetic behavior toward their substrates and their potential as OP bioscavengers.

## Methods

### DNA constructs

Previously, we reported that the full-length human WT BChE gene (UniProt accession number P06276) was optimized for expression in *N*. *benthamiana* plants (pBChE)^8, 9^. A synthetic gene encoding the A328W/Y332A mutant of pBChE^14, 25, 58^ (named here pBChE_V1_) was used as the template for successive rounds of site-directed mutagenesis using the QuickChange method (Stratagene; mutagenic primers are listed in Supplementary Table S2) yielding the following mutants: Variant 2 (pBChE_V2_): F227A/S287G/A328W/Y332A^59^, Variant 3 (pBChE_V3_): A199S/S287G/A328W/Y332G^15^, Variant 4 (pBChE_V4_): A199S/F227A/S287G/A328W/Y332G^18^, and Variant 5 (pBChE_V5_): F227A/S287G/A328W/Y332G (Brimijoin and co-workers, unpublished). The mutated genes (also listed in Supplementary Table S1) were verified by DNA sequencing. A C-terminal hexahistidine tag was added to the plant-expression optimized variants of BChE. The resulting constructs were then cloned into a deconstructed tobacco mosaic virus (TMV)-based plant expression vector (MagnICON, kind gift of Nomad Inc.) to be used in *Agrobacterium tumefaciens*-mediated transient expression in *N*. *benthamiana*.

### Transient recombinant protein production in plants

An outline of the expression strategy is shown in Fig. 1a. All expression vectors were electroporated into *A*. *tumefaciens* strain GV3101 electro-competent cells. Transformed strains were screened via antibiotic selection as well as colony screen PCR and only positive colonies were used for downstream studies. Bacteria cultures were grown at 30 °C until mid-logarithmic phase, pelleted by centrifugation at 4,500 × *g* for 20 min at room temperature and then resuspended in infiltration buffer (10 mM 2-(N-morpholino)ethanesulfonic acid (MES), 10 mM magnesium sulfate heptahydrate, pH 5.5). Plants were infected either by needle-less syringe injection or by whole-plant vacuum infiltration. Leaves infiltrated with each variant were harvested at the respective day of peak expression as determined in previous reports^24^.

### Enzymatic assays

To evaluate cocaine hydrolysis, a sensitive radiometric assay was used as previously described^60^. Briefly, [^3^H](−)-cocaine labeled on the benzene ring (50 Ci/mmol), purchased from PerkinElmer Life Sciences (Boston, MA), was used as a substrate with varying concentrations of (−)-cocaine. In the presence of enzyme this reaction proceeded at room temperature (25**°**C) until stopped by the addition of 0.02 M HCl. Any neutralized, liberated, labeled benzoic acid was then extracted with a toluene-based fluor and measured by scintillation counting. On the other hand, the substrate would fractionate into the aqueous phase and would not generate scintillation. Enzyme concentrations in the reaction mix were 800 ng/100 µL (1.21 × 10^-1^ µM) for WT pBChE and 4 ng/100 µL (6.06 × 10^−4^ µM) for pBChE_V4_.

Choline ester hydrolysis activity was evaluated by a modified Ellman assay^8, 61, 62^. Activity was measured using either butyrylthiocholine iodide (BTC, Sigma) or acetylthiocholine iodide (ATC, Sigma) at 30 °C in a Spectramax 190 spectrophotometer (Molecular Devices). Total soluble protein levels were determined by the Bradford protein assay (Bio-Rad Protein Assay Reagent, Bio-Rad)^63^. The assay was conducted in 96-well plate format over varying concentrations of BTC or ATC in final well volume of 200 μL. To account for product formed by substrate self-hydrolysis, initial velocity of non-enzymatic hydrolysis was subtracted from initial velocity of the matched enzyme-catalyzed reactions and reaction rates were then plotted as a function of substrate concentration.

Data were plotted using GraphPad Prism software, which was also used to fit the data by non-linear regression. The following models were fitted.

For Michaelis-Menten kinetics we used Equation (1).

For substrate inhibition/activation, we used Equation (2), following the model in Scheme 1 (Supplementary Fig. S1), as was suggested by Radić and coworkers^39^.

The Radić model^39^ ascribes the allosteric effect of substrate binding at the peripheral binding site that causes a change in the catalytic rate *bk*_*cat*_. When b > 1 we encounter substrate activation (WT BChE, see below), when b < 1 we encounter substrate inhibition and when b = 1 we have a Michaelian enzyme (Equation 1). K_ss_ is the dissociation constant of the peripheral site.

Velocity *vs* substrate concentration data of some of the BChE variants described here fitted well to a model initially suggested by LiCata and Allewell^64^ for aspartate transcarbamylase (Scheme 2 in Supplementary Fig. S1). This model describes the reaction in terms of uncompetitive substrate inhibition/activation and cooperative substrate binding with characteristic Hill coefficients. The equation describing this model is Equation (3).

The Hill coefficients n and x need not be integers. Values greater than one describe cooperativity, while values of less than one describe anti-cooperativity. The parameters *b* and K_ss_ function in the same way as in Equation (2).

1 $$v=(\frac{{V}_{max}[S]}{{K}_{M}+[S]})$$

2 $$v=(\frac{1+b[S]/{K}_{ss}}{1+[S]/{K}_{ss}})(\frac{{V}_{max}}{1+{K}_{M}/[S]})$$

3 $$v=\frac{{V}_{max}(1+b{[S]}^{x}/{K}_{ss}^{x})}{1+({K}_{M}^{n}/{[S]}^{n})+({[S]}^{x}/{K}_{ss}^{x})}$$

### Inhibition

Inhibition studies were conducted with the OPs paraoxon (diethyl (4-nitrophenyl) phosphate) and iso-OMPA (N- [bis(propan-2-ylamino)phosphoryloxy-(propan-2-ylamino)phosphoryl]propan-2-amine), the carbamate neostigmine bromide ([3-(dimethylcarbamoyloxy)phenyl]-trimethylazanium;bromide), or the reversible bisquaternary inhibitor BW284c51 (BW, [4-[5-[4-[dimethyl(prop-2-enyl)azaniumyl]phenyl]-3-oxopentyl]phenyl]-dimethyl-prop-2-enylazanium;dibromide). The four inhibitors were purchased from Sigma (St Louis, MO).

Preparations of BChE and variants thereof were incubated in 96-well plate format with indicated concentrations of the inhibitors for 30 min at room temperature followed by activity measurements based on modified Ellman assay using 1 mM BTC as the substrate. IC_50_ values were determined by non-linear regression (GraphPad Prism) fit according to Equation (4).

The inhibition rate constant (k_i_) of pBChE_V4_ treated with paraoxon was determined as previously described^65^.

Inhibition curves were statistically analyzed by the extra sum-of-squares F test (GraphPad Prism) together and were found to be significantly different from each other. Following up with individual comparisons to WT pBChE revealed statistical significance in all except the following: paraoxon inhibition of WT pBChE *vs* WT hBChE, Iso-OMPA inhibition of WT pBChE *vs* pBChE_V4_, and neostigmine inhibition of WT pBChE *vs* pBChE_V3_.

4 $$\text{residual}\,\text{BChE}\,\text{activity}\,=\,\frac{100}{1+{10}^{(log[I]-logI{C}_{50})}}$$

### Dynamic Flexibility Index (*DFI*) analysis

Dynamic flexibility index (*DFI*) metric^51^ is based on the Perturbation Response Scanning method (PRS) that couples covariance matrix of residue displacement with linear response theory (LRT)^49, 66, 67^.

PRS was originally based on the Elastic Network Model (ENM). In ENM, protein is viewed as an elastic network, in which each amino acid is represented by C-alpha position, and a harmonic interaction is assigned to pairs of amino-acids within a specified cutoff distance^67^. Simply put, in the WT BChE, two residues that are interacting with each other are represented by a harmonic interaction with the same spring constant (lower left in Supplementary Fig. S2). In contrast, a mutation at a given position is considered to destabilize the interactions of the mutational site. Thus, this destabilization is introduced in the ENM as a decrease in spring constant providing a loss in interaction strength with mutated positions (lower right in Supplementary Fig. S2), the mutation positions S199, A227, G287, W328, and G332 are shown as red spheres).

In PRS, we apply a random Brownian kick as a perturbation to a single residue in the chain one at a time, sequentially. This perturbation mimics the external forces exerted on the protein through interactions with another protein, another biological macromolecule or small molecule ligand, *in silico*. The perturbation cascades through the residue interaction network and may introduce conformational changes in the protein. Then, we compute the response fluctuation profile of all other residues to the perturbation as linear response using Equation (5) where F is a unit random force on selected residues, *H* ^−1^ is the inverse of the Hessian matrix and ΔR is the positional displacements of the N residues of the protein in three dimensions^50, 51, 66–68^.

The response fluctuation profile is used to calculate the *DFI* scores using Equation (6), where $\lceil {\rm{\Delta }}{R}^{j}{\rceil }_{i}$ is the response fluctuation amplitude of position i, upon perturbing position j. Thus, the *DFI* of position i is the total fluctuation response of position i upon perturbing all positions in the chain one at a time. The *DFI* value for each position is normalized to the overall intrinsic flexibility of the protein chain. High and low *DFI* scores could be interpreted as dynamically flexible sites and rigid (hinge) sites, respectively^55^. The *DFI* scores can be converted into percentile ranking scores, namely % *DFI*.

5 $${[{\rm{\Delta }}R]}_{3Nx1}={[H]}_{3Nx3N}^{-1}{[{\rm{F}}]}_{3Nx1}$$

6 $$DF{I}_{i}=\frac{{\sum }_{j=1}^{N}\lceil {\rm{\Delta }}{R}^{j}{\rceil }_{i}}{{\sum }_{i=1}^{N}{\sum }_{j=1}^{N}\lceil {\rm{\Delta }}{R}^{j}{\rceil }_{i}}$$

### Dynamic Coupling Index (DCI) Analysis

The position-specific metric *DCI* uses PRS methodology to identify the residues that are allosterically linked to functionally critical positions through residue fluctuation dynamics for a given protein. The index *DCI* computes whether this position exhibits a higher fluctuation response to a perturbation that occurred at functionally critical sites (e.g. binding sites or catalytic site) compared to the perturbations at the other sites of the chain. It is measured as the ratio of average fluctuation responses of a given residue *j* upon perturbations of functionally critical sites to the average response of residue *j* upon perturbations placed on all other residues using

7 $$\,DC{I}_{i}=\frac{{\sum }_{j={N}_{{\rm{functional}}}}^{{N}_{{\rm{functional}}}}{|{\rm{\Delta }}{R}^{j}|}_{i}/{N}_{{\rm{functional}}}}{{\sum }_{i=1}^{N}{\sum }_{j=1}^{N}{|{\rm{\Delta }}{R}^{j}|}_{i}/N}$$

In Equation (7), ${|{\rm{\Delta }}{R}^{j}|}_{i}$ is the response fluctuation profile of residue *j* upon perturbation of residue *i*. The numerator is the average mean square fluctuation response obtained over the perturbation of the functionally critical residues *N*_*functional*_; the denominator is the average mean square fluctuation response over all residues^49, 50^.

These *DCI* profiles can also be converted into rank profiles, which are labeled as % *DCI* profiles. The positions that have higher % *DCI* values are functionally important residues that are not linked to the functional residues by direct covalent bonds or non-covalent interactions (e.g. hydrogen bonding and van der Waals interactions), but are allosterically communicating over longer distances (i.e. allosteric dynamic coupling) via residues that form extensive interaction networks.

### Purification

All extraction and purification procedures were carried out at 4 °C. Large-scale protein preparations were extracted from plant leaf tissue by blending in the presence of 50 mM sodium phosphate, 150 mM sodium metabisulfite, 1 mM EDTA, pH 8.0. Extract was filtered through double-layer miracloth and centrifuged at 22,000 × *g* for 30 min followed by pH adjustment to pH 5.0 and further clarification by ammonium sulfate precipitation. The pellet was resuspended in cold 1X phosphate buffered saline (PBS) and dialyzed overnight against 1X PBS, pH 7.4 to remove salts and sodium metabisulfite. The clarified protein preparation was then subjected to sequential affinity chromatography steps with Concanavalin-A-Sepharose followed by procainamide affinity chromatography as previously described^8^.

### SDS-PAGE and western blot

Plant-derived protein preparations were resolved by SDS-PAGE on 8% polyacrylamide gels followed by staining with Pierce Silver Stain Kit (Thermoscientific). In parallel, protein was transferred to nitrocellulose membrane and decorated with rabbit polyclonal anti-hBChE antibodies (kindly provided by Dr. Oksana Lockridge) and anti-rabbit IgG-Horse Radish Peroxidase secondary antibodies (Santa Cruz Biotechnology) followed by chemiluminescence analysis using western blotting luminol reagent (Santa Cruz Biotechnology).

### Size exclusion HPLC

SEC-HPLC fractionation of purified preparations of pBChE_V4_ was carried out as previously described using Alliance HPLC (Waters) with a Shodex KW-803 column (8 × 300 mm, Kawasaki)^8^. All samples were run in filtered, degassed mobile phase buffer (20 mM Na_2_HPO_4_/NaH_2_PO_4_, pH 8.0, 200 mM NaCl, 0.04% NaN_3_) at a flow rate of 0.5 mL/min. Molecular mass standards used were blue dextran (2000 kDa) and the proteins β-amylase (200 kDa), bovine serum albumin (66 kDa) and carbonic anhydrase (29 kDa). Fractions were collected and analyzed for cholinesterase activity by the modified Ellman assay.

### Data availability statement

All data generated or analyzed during this study are included in this published article (and its Supplementary Information files) except for the raw computational datasets that are available from the corresponding author on reasonable request.

## Electronic supplementary material

Supplemental Tables, Figure Legends and Figures

## References

[1] Lockridge O (2015). Review of human butyrylcholinesterase structure, function, genetic variants, history of use in the clinic, and potential therapeutic uses. Pharmacol Ther.

[2] Khan SB (2005). Butyrylcholinesterase inhibitory guaianolides from Amberboa ramosa. Arch Pharm Res.

[3] Decker M (2005). Novel inhibitors of acetyl- and butyrylcholinesterase derived from the alkaloids dehydroevodiamine and rutaecarpine. Eur J Med Chem.

[4] Loizzo MR, Tundis R, Menichini F (2008). Natural products and their derivatives as cholinesterase inhibitors in the treatment of neurodegenerative disorders: an update. Curr Med Chem.

[5] Chen VP (2015). Plasma butyrylcholinesterase regulates ghrelin to control aggression. Proc Natl Acad Sci USA.

[6] Saxena A (2015). Prophylaxis with human serum butyrylcholinesterase protects Gottingen minipigs exposed to a lethal high-dose of sarin vapor. Chem Biol Interact.

[7] Doctor BP, Saxena A (2005). Bioscavengers for the protection of humans against organophosphate toxicity. Chem Biol Interact.

[8] Geyer BC (2010). Transgenic plants as a source for the bioscavenging enzyme, human butyrylcholinesterase. Plant Biotechnol J.

[9] Geyer BC (2010). Plant-derived human butyrylcholinesterase, but not an organophosphorous-compound hydrolyzing variant thereof, protects rodents against nerve agents. Proc Natl Acad Sci USA.

[10] Inaba T, Stewart DJ, Kalow W (1978). Metabolism of cocaine in man. Clin Pharmacol Ther.

[11] Carmona GN (2000). Butyrylcholinesterase accelerates cocaine metabolism: *in vitro* and *in vivo* effects in nonhuman primates and humans. Drug Metab Dispos.

[12] Zheng F, Zhan CG (2011). Enzyme-therapy approaches for the treatment of drug overdose and addiction. Future Med Chem.

[13] Xie W (1999). An improved cocaine hydrolase: the A328Y mutant of human butyrylcholinesterase is 4-fold more efficient. Mol Pharmacol.

[14] Sun H, Pang YP, Lockridge O, Brimijoin S (2002). Re-engineering butyrylcholinesterase as a cocaine hydrolase. Mol Pharmacol.

[15] Pan Y (2005). Computational redesign of human butyrylcholinesterase for anticocaine medication. Proc Natl Acad Sci USA.

[16] Zheng F (2008). Most efficient cocaine hydrolase designed by virtual screening of transition states. J Am Chem Soc.

[17] Xue L (2011). Design, preparation, and characterization of high-activity mutants of human butyrylcholinesterase specific for detoxification of cocaine. Mol Pharmacol.

[18] Xue L (2013). Preparation and *in vivo* characterization of a cocaine hydrolase engineered from human butyrylcholinesterase for metabolizing cocaine. Biochem J.

[19] Zheng F (2014). A highly efficient cocaine-detoxifying enzyme obtained by computational design. Nat Commun.

[20] Chen X (2016). Long-acting cocaine hydrolase for addiction therapy. Proc Natl Acad Sci USA.

[21] Connors NJ, Hoffman RS (2013). Experimental treatments for cocaine toxicity: a difficult transition to the bedside. J Pharmacol Exp Ther.

[22] Topp E (2016). The case for plant-made veterinary immunotherapeutics. Biotechnol Adv.

[23] Mor TS (2015). Molecular pharming’s foot in the FDA’s door: Protalix’s trailblazing story. Biotechnol Lett.

[24] Larrimore KE (2013). Plants as a source of butyrylcholinesterase variants designed for enhanced cocaine hydrolase activity. Chem Biol Interact.

[25] Geyer BC, Woods RR, Mor TS (2008). Increased organophosphate scavenging in a butyrylcholinesterase mutant. Chem Biol Interact.

[26] Sun H (2001). Predicted Michaelis-Menten complexes of cocaine-butyrylcholinesterase. Engineering effective butyrylcholinesterase mutants for cocaine detoxication. J Biol Chem.

[27] Zlebnik NE (2014). Long-term reduction of cocaine self-administration in rats treated with adenoviral vector-delivered cocaine hydrolase: evidence for enzymatic activity. Neuropsychopharmacology.

[28] Xue L (2013). Catalytic activities of a cocaine hydrolase engineered from human butyrylcholinesterase against (+)- and (−)-cocaine. Chemico-Biological Interactions.

[29] Zheng F (2010). Design of high-activity mutants of human butyrylcholinesterase against (-)-cocaine: structural and energetic factors affecting the catalytic efficiency. Biochemistry.

[30] Yang W, Xue L, Fang L, Chen X, Zhan CG (2010). Characterization of a high-activity mutant of human butyrylcholinesterase against (−)-cocaine. Chem Biol Interact.

[31] Gao D (2006). Computational design of a human butyrylcholinesterase mutant for accelerating cocaine hydrolysis based on the transition-state simulation. Angew Chem Int Ed Engl.

[32] Gao D, Zhan CG (2006). Modeling evolution of hydrogen bonding and stabilization of transition states in the process of cocaine hydrolysis catalyzed by human butyrylcholinesterase. Proteins.

[33] Schneider JD (2014). Expression of human butyrylcholinesterase with an engineered glycosylation profile resembling the plasma-derived orthologue. Biotechnol J.

[34] Zhan M, Hou S, Zhan CG, Zheng F (2014). Kinetic characterization of high-activity mutants of human butyrylcholinesterase for the cocaine metabolite norcocaine. Biochem J.

[35] Masson P, Xie W, Froment MT, Lockridge O (2001). Effects of mutations of active site residues and amino acids interacting with the Omega loop on substrate activation of butyrylcholinesterase. Biochim Biophys Acta.

[36] Barak D (1995). Allosteric modulation of acetylcholinesterase activity by peripheral ligands involves a conformational transition of the anionic subsite. Biochemistry.

[37] Boeck AT, Schopfer LM, Lockridge O (2002). DNA sequence of butyrylcholinesterase from the rat: expression of the protein and characterization of the properties of rat butyrylcholinesterase. Biochem Pharmacol.

[38] Chen X, Fang L, Liu J, Zhan CG (2012). Reaction pathway and free energy profiles for butyrylcholinesterase-catalyzed hydrolysis of acetylthiocholine. Biochemistry.

[39] Radic Z, Pickering NA, Vellom DC, Camp S, Taylor P (1993). Three distinct domains in the cholinesterase molecule confer selectivity for acetyl- and butyrylcholinesterase inhibitors. Biochemistry.

[40] Evron T (2007). Plant-derived human acetylcholinesterase-R provides protection from lethal organophosphate poisoning and its chronic aftermath. Faseb J.

[41] Shafferman A (1992). Substrate inhibition of acetylcholinesterase: residues affecting signal transduction from the surface to the catalytic center. EMBO J..

[42] Chen X (2015). Kinetic characterization of a cocaine hydrolase engineered from mouse butyrylcholinesterase. Biochem J.

[43] Velan B (1991). The effect of elimination of intersubunit disulfide bonds on the activity, assembly, and secretion of recombinant human acetylcholinesterase. Expression of acetylcholinesterase Cys-580-Ala mutant. J Biol Chem.

[44] Blong RM, Bedows E, Lockridge O (1997). Tetramerization domain of human butyrylcholinesterase is at the C-terminus. The Biochemical journal.

[45] Saxena A, Hur RS, Luo C, Doctor BP (2003). Natural monomeric form of fetal bovine serum acetylcholinesterase lacks the C-terminal tetramerization domain. Biochemistry.

[46] Schneider JD (2014). Oligomerization status influences subcellular deposition and glycosylation of recombinant butyrylcholinesterase in Nicotiana benthamiana. Plant Biotechnol J.

[47] Rosenberry TL (2010). Strategies to resolve the catalytic mechanism of acetylcholinesterase. J Mol Neurosci.

[48] Felder CE, Harel M, Silman I, Sussman JL (2002). Structure of a complex of the potent and specific inhibitor BW284C51 with Torpedo californica acetylcholinesterase. Acta Crystallogr D Biol Crystallogr.

[49] Kumar A, Glembo TJ, Ozkan SB (2015). The Role of Conformational Dynamics and Allostery in the Disease Development of Human Ferritin. Biophys J.

[50] Gerek ZN, Ozkan SB (2011). Change in allosteric network affects binding affinities of PDZ domains: analysis through perturbation response scanning. PLoS Comput Biol.

[51] Nevin Gerek Z, Kumar S, Banu Ozkan S (2013). Structural dynamics flexibility informs function and evolution at a proteome scale. Evol Appl.

[52] Butler BM, Gerek ZN, Kumar S, Ozkan SB (2015). Conformational dynamics of nonsynonymous variants at protein interfaces reveals disease association. Proteins.

[53] Kim H (2015). A hinge migration mechanism unlocks the evolution of green-to-red photoconversion in GFP-like proteins. Structure.

[54] Zou T, Risso VA, Gavira JA, Sanchez-Ruiz JM, Ozkan SB (2015). Evolution of conformational dynamics determines the conversion of a promiscuous generalist into a specialist enzyme. Mol Biol Evol.

[55] Kumar A, Butler BM, Kumar S, Ozkan SB (2015). Integration of structural dynamics and molecular evolution via protein interaction networks: a new era in genomic medicine. Curr Opin Struct Biol.

[56] Alvarez-Garcia D, Barril X (2014). Relationship between Protein Flexibility and Binding: Lessons for Structure-Based Drug Design. J Chem Theory Comput.

[57] Li Z (2015). A Rigid Hinge Region Is Necessary for High-Affinity Binding of Dimannose to Cyanovirin and Associated Constructs. Biochemistry.

[58] Sun H, Shen ML, Pang YP, Lockridge O, Brimijoin S (2002). Cocaine metabolism accelerated by a re-engineered human butyrylcholinesterase. J Pharmacol Exp Ther.

[59] Pancook JD (2003). Application of directed evolution technology to optimize the cocaine hydrolase activity of human butyrylcholinesterase. Faseb Journal.

[60] Brimijoin S, Shen ML, Sun H (2002). Radiometric solvent-partitioning assay for screening cocaine hydrolases and measuring cocaine levels in milligram tissue samples. Anal Biochem.

[61] Geyer BC (2005). Purification of Transgenic Plant-Derived Recombinant Human Acetylcholinesterase-R. Chem Biol Interact.

[62] Geyer BC (2007). Translational control of recombinant human acetylcholinesterase accumulation in plants. BMC Biotechnol.

[63] Mor TS, Sternfeld M, Soreq H, Arntzen CJ, Mason HS (2001). Expression of recombinant human acetylcholinesterase in transgenic tomato plants. Biotechnol. Bioeng..

[64] LiCata VJ, Allewell NM (1997). Is substrate inhibition a consequence of allostery in aspartate transcarbamylase?. Biophys Chem.

[65] Mionetto N, Morel N, Massoulie J, Schmid RD (1997). Biochemical determination of insecticides via cholinesterases .1. Acetylcholinesterase from rat brain: Functional expression using a baculovirus system, and biochemical characterization. Biotechnology Techniques.

[66] Atilgan C, Atilgan AR (2009). Perturbation-response scanning reveals ligand entry-exit mechanisms of ferric binding protein. PLoS Comput Biol.

[67] Atilgan C, Gerek ZN, Ozkan SB, Atilgan AR (2010). Manipulation of conformational change in proteins by single-residue perturbations. Biophys J.

[68] Atilgan AR (2001). Anisotropy of fluctuation dynamics of proteins with an elastic network model. Biophys J.

