# Author Correction: Plant-expressed cocaine hydrolase variants of butyrylcholinesterase exhibit altered allosteric effects of cholinesterase activity and increased inhibitor sensitivity

**DOI:** 10.1038/s41598-018-35441-0

**Published:** 2018-11-16

**Authors:** Katherine E. Larrimore, I. Can Kazan, Latha Kannan, R. Player Kendle, Tameem Jamal, Matthew Barcus, Ashini Bolia, Stephen Brimijoin, Chang-Guo Zhan, S. Banu Ozkan, Tsafrir S. Mor

**Affiliations:** 1 School of Life Sciences and Center for Immunotherapy, Vaccines, and Virotherapy, Biodesign Institute, Arizona State University, Tempe, AZ 85287-4501 USA; 2 Department of Physics and Center for Biological Physics, Arizona State University, Tempe, AZ 85287-1504 USA; 3 Department of Molecular Pharmacology and Experimental Therapeutics, Mayo Clinic, Rochester, MN 55905 USA; 4 Molecular Modeling and Biopharmaceutical Center and Department of Pharmaceutical Sciences, College of Pharmacy, University of Kentucky, Lexington, KY 40536 USA; 5 Present Address: Temasek Life Sciences Laboratory, National University of Singapore, Singapore, 117604 Singapore; 6 Present Address: Department of Botany, College of Letters and Sciences, University of Wisconsin Madison, Madison, WI 53706 USA; 7 Present Address: Department of Animal Science, College of Agriculture and Life Sciences, Cornell University, Ithaca, NY 14853 USA; 8 Present Address: ARUP Labs, Salt Lake City, UT 84108 USA

Correction to: *Scientific Reports* 10.1038/s41598-017-10571-z, published online 05 September 2017

This Article contains an error in Equation 7.

$${{\rm{DCI}}}_{i}=\frac{{\sum }_{j={N}_{functional}}^{{N}_{functional}}|{\rm{\Delta }}{R}^{j}{|}_{i}/{N}_{functional}}{{\sum }_{i=1}^{N}{\sum }_{j=1}^{N}|{\rm{\Delta }}{R}^{j}{|}_{i}/N}$$

should read:

$${{\rm{DCI}}}_{i}=\frac{{\sum }_{j={N}_{functional}}^{{N}_{functional}}|{\rm{\Delta }}{R}^{j}{|}_{i}/{N}_{functional}}{{\sum }_{j=1}^{N}|{\rm{\Delta }}{R}^{j}{|}_{i}/N}$$

