# Supplementary material for: Plant-expressed cocaine hydrolase variants of butyrylcholinesterase exhibit altered allosteric effects of cholinesterase activity and increased inhibitor sensitivity
Source: Sci Rep. 2017 Sep 5;7:10419. doi: 10.1038/s41598-017-10571-z (PMC5585256; doi:10.1038/s41598-017-10571-z)

SUPPLEMENTARY INFORMATION

Plant-expressed cocaine hydrolase variants of butyrylcholinesterase exhibit altered allosteric effects of cholinesterase activity and increased inhibitor sensitivity.

Katherine E Larrimore[1,†], I. Can Kazan[2], Latha Kannan[1], R. Player Kendle[1,‡], Tameem Jamal[1], Matthew Barcus[1,^], Ashini Bolia[2,¥], Stephen Brimijoin[3], Chang-Guo Zhan[4], S. Banu Ozkan[2], and Tsafrir S Mor[1,*]

[1]School of Life Sciences and Center of Immunotherapy, Vaccines, and Virotherapy, Biodesign Institute, Arizona State University, Tempe, AZ 85287-4501, USA

[2]Department of Physics and Center for Biological Physics, Arizona State University Tempe, AZ 85287-1504, USA

[3]Department of Molecular Pharmacology and Experimental Therapeutics, Mayo Clinic, Rochester, MN 55905, USA

[4]Molecular Modeling and Biopharmaceutical Center and Department of Pharmaceutical Sciences, College of Pharmacy, University of Kentucky, Lexington, KY 40536, USA

[†]Current Address: Temasek Life Sciences Laboratory, National University of Singapore, Singapore 117604

[‡]Current Address: Department of Botany, College of Letters and Sciences, University of Wisconsin Madison , Madison , WI 53706, USA

[^]Current Address: Department of Animal Science, College of Agriculture and Life Sciences, Cornell University, Ithaca, NY 14853, USA

¥ Current Address: ARUP Labs, Salt Lake City, UT 84108, USA

*To whom correspondence should be addressed:

School of Life Sciences and The Biodesign Institute

P.O. Box 874501

Arizona State University

Tempe, AZ 85287-4501, USA

Tel: 1-480-727-7405; Fax: 1- 480-965-6899

Email: tsafrir.mor@asu.edu

Supplementary material includes:

1. Figure Legends for Supplementary Figures S1 and S2
2. Supplementary Table S1 and Table S2
3. Supplementary Figures S1 and S2

Legends for Supplementary Figures

Supplementary Figure S1. Schematic diagrams describing the kinetics of cholinesterase-catalyzed hydrolysis of substrates

(**a**) Scheme 1 describes the reaction of cholinesterase (E)-catalyzed hydrolysis of substrates (S). K_{ss} is the dissociation constant of the peripheral site. The hydrolysis capacity (bK_{cat}) reflects the allosteric effect of substrate binding at the peripheral binding site. (**b**) Scheme 2 describes the reaction in terms of uncompetitive substrate inhibition/activation and cooperative substrate binding with characteristic Hill coefficients n and x that describe cooperativity or anticooperativity

Supplementary Figure S2. Modeling of wild type and mutant by using Elastic Network Model (ENM)

WT human BChE and $BChE_{V4}$ are modeled by ENM. The spheres indicate the locations of alpha-carbons of each amino acid and the sticks are representing the harmonic oscillators (i.e. springs) between them. The thickness of the sticks represents the magnitude of the spring constant. For WT hBChE the mutation positions are shown as blue spheres (A199, F227, S287, A328, and Y332) and for the pentavalent mutant $BChE_{V4}$ the mutation positions are shown as red spheres (S199, A227, G287, W328, and G332). The spring constant for each connection is assumed to be same for the WT (i.e. the thickness of the blue sticks is same as grey sticks). The mutation at a given position are considered to destabilize the interactions of the mutational site. This is incorporated in the model as a decrease in spring constant (low thickness values are shown as red sticks indicating a loss in interaction strength with mutated positions).

Supplementary Table S1

Table S1. Cocaine hydrolase variants of butyrylcholinesterase used in this study.

Name	Amino acid mutations	References
$pBChE_{V2}$	F227A/S287G/A328W/Y332A	Pancook, *et al.* 2003
$pBChE_{V3}$	A199S/S287G/A328W/Y332G	Pan, *et al.* 2005
$pBChE_{V4}$	A199S/F227A/S287G/A328W/Y332G	Xue, *et al.* 2013
$pBChE_{V5}$	F227A/S287G/A328W/Y332G	Brimijoin and co-workers, unpublished

Supplementary Table S2

Table S2. Oligonucleotides used for site-directed mutagenesis. Forward (F) and reverse (R) primers are shown with mutation sites indicated in lowercase.

Name	Primer Sequence	Mutation
oTM607	(F) $^{5'}$CTTTGGAGAGTCTtctGGAGCTGCTTCTG$^{3'}$	A199S
oTM608	(R) $^{5'}$CAGAAGCAGCTCCagaAGACTCTCCAAAG$^{3'}$	A199S
oTM609	(F) $^{5'}$CCAATCTGGTTCCgctAATGCTCCTTGG$^{3'}$	F227A
oTM610	(R) $^{5'}$CCAAGGAGCATTagcGGAACCAGATTGG3'	F227A
oTM611	(F) $^{5'}$GGAACTCCTTTGggaGTGAACTTTGGTC$^{3'}$	S287G
oTM612	(R) $^{5'}$GACCAAAGTTCACtccCAAAGGAGTTCC$^{3'}$	S287G
oTM613	(F) $^{5'}$GGATGAGGGTACAtggTTCCTTGTGggtGGAGCGCCTGG$^{3'}$	A328W/Y332G
oTM614	(R) $^{5'}$CCAGGCGCTCCaccCACAAGGAAccaTGTACCCTCATCC$^{3'}$	A328W/Y332G
oTM655	(F) $^{5'}$GGTTCCTTGTGgctGGAGCGCCTGG$^{3'}$	Y332A
oTM656	(R) $^{5'}$CCAGGCGCTCCagcCACAAGGAACC$^{3'}$	Y332A

Supplementary Figure S1

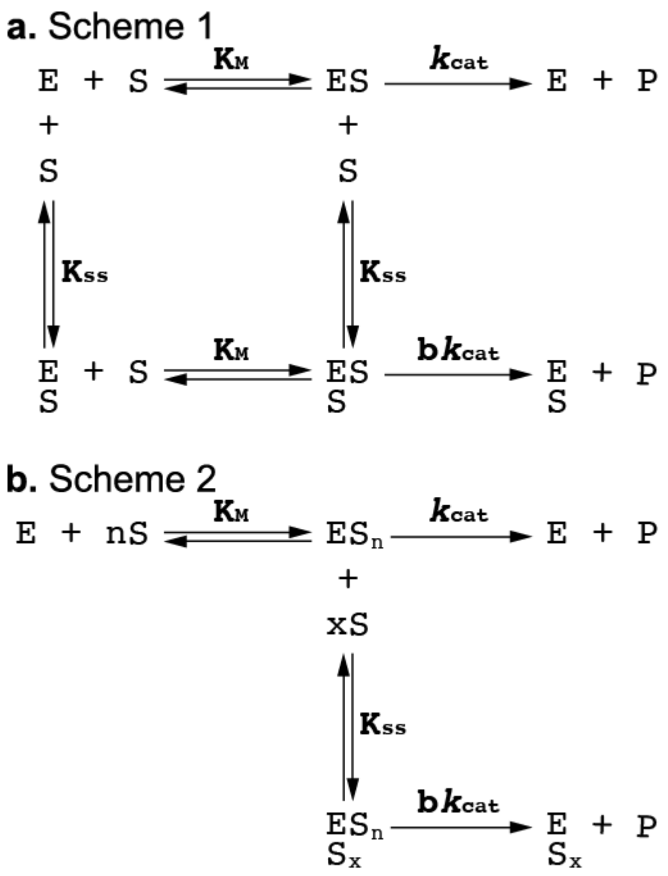

Supplementary Figure S2

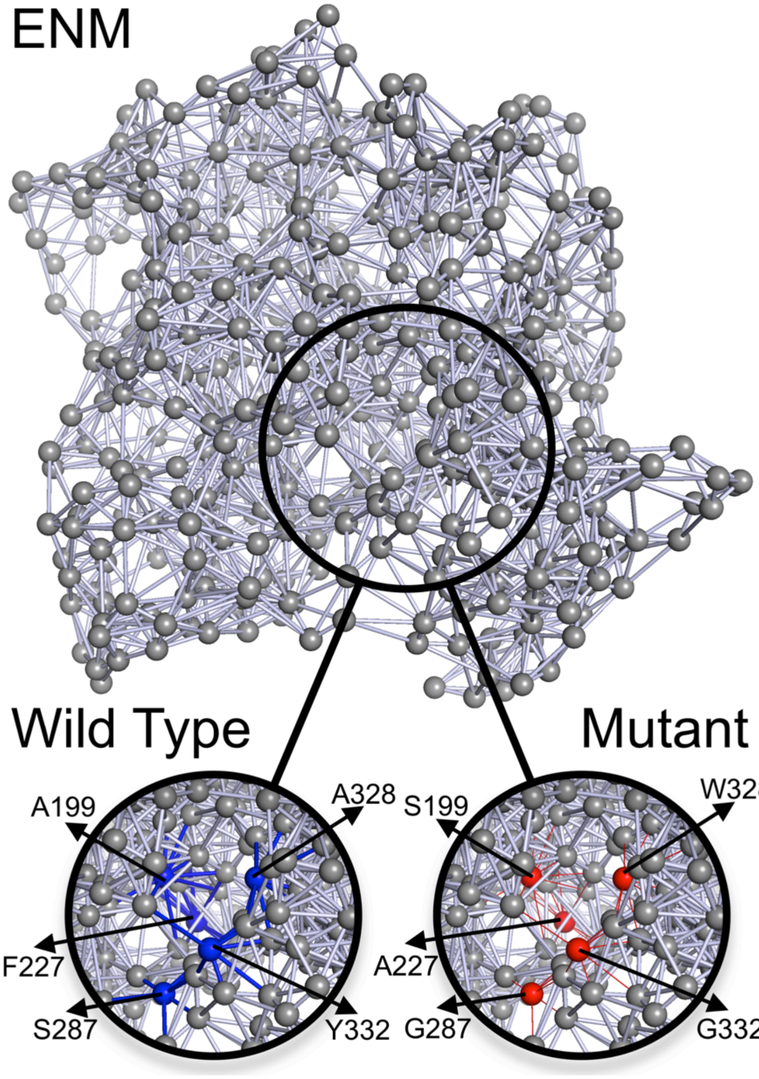

Supplement: Supplementary file 1 — Supplemental Tables, Figure Legends and Figures [file 41598_2017_10571_MOESM1_ESM.pdf]
